# Supplementary material for: Update on Findings about Sudden Sensorineural Hearing Loss and Insight into Its Pathogenesis
Source: J Clin Med. 2022 Oct 28;11(21):6387. doi: 10.3390/jcm11216387 (PMC9653771; doi:10.3390/jcm11216387)
Supplement: Supplementary file 1 [file jcm-11-06387-s001.zip › jcm-1968394-supplementary.pdf]

Table S1. Summarized findings of the studies included for serological examination.

Authors (year)	Study Design	Study Methods	Results
Ulu S et al. (2013) [16]	retrospective	Forty-seven ISSNHL patients and 45 age- and sex-matched healthy subjects were enrolled. NLR measurements and hearing assessments were carried out.	The mean NLR, neutrophil, and lymphocyte values in patients with ISSNHL were significantly higher than in the control group.
Qiao X-F et al. (2019) [17]	retrospective	Sixty SSNHL and 60 healthy volunteers were enrolled. NLR and PLR were compared between these groups.	The average NLR and PLR of SSNHL patients were both significantly higher than in the control group.
Kuzucu İ et al. (2020) [21]	prospective	Forty-four ISSNHL patients and 41 healthy volunteers were enrolled.The relationship between the groups was statistically evaluated for serum calprotectin levels.	Serum calprotectin value in the severe group was significantly higher compared to the moderate and mild groups. Serum calprotectin value in the recovered group was significantly lower compared to the partially recovered and unrecovered groups.
Yoon SH et al. (2019) [22]	retrospective	Twenty-four SSNHL patients and 24 healthy volunteers were enrolled. PBMC, CD11c, and CD86 were isolated and analyzed. Plasma and supernatant levels of TNFα, IFNγ, and IL10 and IL12 were measured.	Monocyte population, mean TNFα level, and CD86 expression were significantly increased in the SSNHL group. IFNγ and IL12 levels were significantly decreased. The difference in mean IL10 level was not significant.
Demirhan E et al. (2013) [23]	prospective	Twenty-three ISSNHL patients and 20 healthy volunteers were enrolled. Blood samples for TNFα, IL10, and IL12 were taken before treatment and 6 weeks after treatment.	There was no significant difference between pre- and post-treatment values of IL10 and IL12. Treatment nonresponders had more elevated posttreatment values of TNFα than pretreatment values.
Bulğurcu S et al. (2017) [26]	retrospective	Twenty-one ISSNHL patients younger than 19 years and 24 healthy volunteers were enrolled. Patients were divided into two groups: those who recovered after treatment and those who did not. NLR and PLR values were compared between these groups.	A statistical significance for the NLR was detected between the patient group and control group and between patients who recovered and those who did not. Whereas, no statistical significance for the PLR was detected.
Lin H-C et al. (2015) [30]	retrospective	One hundred sixty-six ISSNHL patients were enrolled. The ratios of TC/HDL and LDL/HDL were evaluated.	The ratio of LDL/HDL is a prognostic factor for hearing recovery in ISSNHL patients.
Shao M et al. (2021) [31]	prospective	Two hundred thirty-two ISSNHL patients were enrolled. TC, TG, LDL-C, and HDL-C were evaluated.	TC, TG, and the LDL-C/HDL-C ratio are strongly associated with the prognosis of ISSNHL.
Liu Z et al. (2020) [36]	prospective	Forty-seven SSNHL patients and 25 healthy volunteers were enrolled. The expression and activation status of the PERK-C/EBP homologous protein and CHOP pathway in PBMCs were evaluated. Differences in expression of ATF4 and CHOP before and after glucocorticoid treatment were also evaluated.	ERS may play a significant role in the pathogenesis of SSNHL.

CHOP, C/EBP homologous protein; HDL-C, high-density lipoprotein cholesterol; IFNγ, interferon gamma; IL, interleukin; ISSNHL, idiopathic sudden sensorineural hearing loss; LDL-C, low-density lipoprotein-cholesterol; NLR, neutrophil-to-lymphocyte ratio; PBMCs, peripheral blood mononuclear cells; PERK, protein kinase RNA-like ER kinase; PLR, PLR,

platelet-to-lymphocyte ratio; SSNHL, sudden sensorineural hearing loss; TC, total cholesterol; TG, triglyceride; TNFα, tumor necrosis factor alpha.

Table S2. Summarized findings of the studies included for MRI examination.

Authors (year)	Study Design	Study Methods	Results
Berrettini S et al. (2013) [41]	retrospective	Twenty-three ISSNHL patients and 20 healthy volunteers were enrolled. 3D-FLAIR MRI at 3 T were performed.	Thirteen patients showed high-intensity signals in the affected inner ear on precontrast and post-contrast 3D-FLAIR MRI.
Liao W-H et al. (2016) [42]	retrospective	Fifty-four ISSNHL patients were enrolled. MRI with pre contrast FLAIR, post contrast T1WI, and post contrast FLAIR were performed.	Asymmetric cochlear signal intensities were more frequently observed in pre contrast and post contrast FLAIR than in T1WI.
Lee JW et al. (2022) [44]	prospective	One hundred fifteen patients who were diagnosed with SSNHL aged between 55 and 75 years were enrolled. All subjects underwent brain MRI and were divided into a WMH and control groups, depending on the presence of WMH on MRI.	The presence of mild WMH was associated with better treatment response and good prognosis.

FLAIR, fluid-attenuated inversion-recovery; ISSNHL, idiopathic sudden sensorineural hearing loss; MRI, magnetic resonance imaging; SSNHL, sudden sensorineural hearing loss; WMH, white matter hyperintensity.

Table S3. Summarized findings of the studies included for genetic examination.

Authors (year)	Study Design	Study Methods	Results
Capaccio P et at. (2007) [46]	retrospective	One hundred SSNHL patients and 200 healthy individuals were enrolled. Association of SSNHL and SNPs, including *MTHFR* (rs 1801133, and rs 1801131), *F2* (rs1799963), *ITGB3* (rs5918), and *F5* (rs6025), was analyzed.	A significant association was found between SSNHL and SNPs, including *MTHFR* (rs 1801133, and rs 1801131), *F2* (rs1799963), *ITGB3* (rs5918), and *F5* (rs6025).
Görür K et al. (2005) [48]	retrospective	Fifty-six SSNHL patients and 95 healthy individuals were enrolled. Association of SSNHL and SNPs, including *F2* (rs1799963), and *F5* (rs6025), was analyzed.	A significant association was found between SSNHL and *F5* (rs6025).
Ballesteros F et al. (2012) [8]	retrospective	One hudred and eighteen SSNHL patients and 161 healthy individuals were enrolled. Association of SSNHL and SNPs, including *ITGB3* (rs5918), and *ITGA2* (rs1126643), was analyzed.	A significant association was found between SSNHL and *ITGA2* (rs1126643).
Rudack C et al. (2004) [68]	retrospective	Eighty-five SSNHL patients and 85 healthy individuals were enrolled. Association of SSNHL and polymorphisms, including *F5* (rs6025), *F2* (rs1799963), *ITGA2* (rs1126643), *SERPINE1* (rs1799889), *PLAT* (Alu+/Alu−), and *CBS* (c.844ins68), was analyzed.	A significant association was found between SSNHL and *ITGA2* (rs1126643).
Um J-Y et al. (2010) [52]	retrospective	Ninety-seven SSNHL patients and 587 healthy individuals were enrolled. Association of SSNHL and SNPs, including *TNF* (rs1800629), and *LTA* (rs909253), was analyzed.	A significant association was found between SSNHL and *LTA* (rs909253).
Furuta T et al. (2011) [53]	retrospective	Seventy-two SSNHL patients and 2202 healthy individuals were enrolled. Association of SSNHL and	A significant association was found between SSNHL and *IL1A* (rs1800587).

		SNPs, including *IL1A* (rs1800587), and *IL1B* (rs16944), was analyzed.	
Um J-Y et al. (2013) [54]	retrospective	One hundred and two SSNHL patients and 595 healthy individuals were enrolled. Association of SSNHL and SNPs, including *IL1B* (rs16944, and rs1143634), was analyzed.	A significant association was found between SSNHL and SNPs, including *IL1B* (rs16944, and rs1143634)
Nam SI et al. (2006) [55]	retrospective	Ninety-seven SSNHL patients and 613 healthy individuals were enrolled. Association of SSNHL and *IL4R* (rs180275) was analyzed.	A significant association was found between SSNHL and *IL4R* (rs180275).
Tian G et al. (2018) [56]	retrospective	Seventy-five SSNHL patients and 165 healthy individuals were enrolled. Association of SSNHL and SNPs, including *IL6* (rs1800796) and *ICAM1* (rs5498), was analyzed.	A significant association was found between SSNHL and *IL6* (rs1800796). Coexistence of *IL6* (rs1800796) and *ICAM1* (rs5498) was significantly associated with increased SSNHL risk
Cadoni G et al. (2015) [57]	retrospective	Eighty-seven SSNHL patients and 107 hearthy individuals were enrolled. Association of SSNHL and *IL6* (rs1800795) was analyzed.	A significant association was found between SSNHL and *IL6* (rs1800795).
Kitoh R et al. (2016) [69]	retrospective	One hundred and ninety-two SSNHL patients were enrolled. Association of SSNHL and 39 SNPs from 31 genes was analyzed.	A significant association was found between SSNHL and *SOD1* (rs4998557).
Chien C-Y et al. (2017) [60]	retrospective	Four hundred and sixteen SSNHL patients and 255 healthy individuals were enrolled. Association of SSNHL and SNPs, including *GPX3* (rs3763013, rs8177412, rs3805435, rs3828599, and rs2070593), was analyzed.	A significant association was found between SSNHL and *GPX3* (rs3805435).
Castiglione A et al. (2015) [62]	retrospective	Two hundred SSNHL patients and 400 healthy individuals were enrolled. Association of SSNHL and SNPs, including *SLC40A1* (rs11568351), *TF* (rs1049296), *HEF* (rs1799945, rs1800562), and *HAMP* (rs10421768), was analyzed.	A significant association was found between SSNHL and *SLC40A1* (rs11568351).
Nunez DA et al. (2020) [64]	prospective	Thirty-six SSNHL patients and 12 healthy individuals were enrolled. miRNA profiling of 754 human miRNAs was perfomed in SSNHL patients, and was compared with that of the controls.	Eight miRNAs, including hsa-miR-590-5p, hsa-miR-186-5p, hsa-miR-195-5p, hsa-miR-140-3p, hsa-miR-128-3p, hsa-miR-132-3p, hsa-miR-375-3p, and hsa-miR-30a-3p, were identified as significantly differentially expressed in SSNHL patients.

SNP, single-nucleotide polymorphism; SSNHL, sudden sensorineural hearing loss.

Table S4. Summarized findings of the studies included for viral examination.

Authors (year)	Study Design	Study Methods	Results
Cohen BE et al. (2014) [69]	narrative review	Studies on SSNHL caused by viral infections were summarized.	Sensorineural hearing loss occurs during various type of viral infections.
Mateer EJ et al. (2018) [70]	narrative review	Studies on SSNHL caused by Lassa fever were summarized.	The majority of evidence favors the immune-mediated hypothesis, direct Lassa viras damage cannot be ruled out.
Mustafa MWM. (2020) [89]	retrospective	Twenty cases who were confirmed positive for COVID-19 and had none of the known symptoms for this viral infection and 20 healthy volunteers were enrolled. TEOAEs amplitude was measured for all participants.	The high frequency pure-tone thresholds, as well as the TEOAE amplitudes, were significantly worse in the COVID-19 positive group.

Jeong J et al. (2021) [90]	retrospective	Three patients with SSNHL within three days after COVID-19 vaccination were enrolled. Pure tone audiometry and MRI were performed.	No evidence of an association between vaccination and SSNHL. It should be known that SSNHL can occur as an adverse event of COVID-19 vaccination.
Jeong M et al. (2021)[92]	retrospective	Ten COVID-19 patients with audiovestibular symptoms. To investigate the causal relationship between SARS-CoV-2 and audiovestibular dysfunction, human inner ear tissue, human inner ear in vitro cellular models, and mouse inner ear tissue were evaluated.	Adult human inner ear tissues express ACE2 receptor, TMPRSS2, and FURIN cofactors required for virus entry. Study group established three human-induced pluripotent stem cells-derived in vitro models. Inner ear organoids expressed ACE2 receptor.
Jin L et al. (2021)[93]	retrospective	Otolaryngological diseases in the last three years during the same period as the COVID-19 pandemic period (from February to April 2020) were compared.	The number of patients with SSNHL increased during the COVID-19 pandemic.
Parrino D et al. (2022)[94]	retrospective	SSNHL was compared to the COVID-19 pandemic period of March 1, 2020 to February 28, 2021 and two time periods (March 1, 2019 to February 29, 2020 and March 1, 2018 to February 28, 2019).	The number of patients with SSNHL increased during the COVID-19 pandemic.
Fidan V et al. (2021)[95)	retrospective	SSNHL patients in the COVID-19 pandemic period of April 1-September 30, 2020 and the same period in 2019 were compared.	SSNHL incidence increased during the COVID-19 widespread compared to the same interval of the prior year.
Chari D.A et al. (2020)[96]	retrospective	SSNHL patients the March 15-May 31, 2019 before COVID-19 pandemic and the March 15-May 31, 2020 after COVID-19 pandemic were compared.	COVID-19 did not appear to confer a significantly increased risk for the development of SSNHL.
Aslan M et al. (2021)[97]	retrospective	SSNHL patients presenting between April 2020 and April 2021 during the COVID-19 epidemic and between January 2019 and January 2020 before the COVID-19 epidemic were compared.	There was no relationship between COVID-19 and cases of SSNHL.
Doweck I et al. (2022)[98]	retrospective	SSNHL patients were compared between March 1, 2020 after the COVID-19 pandemic and December 19, 2020 until the vaccine was introduced, and the same time period of 2019 and 2018.	SSNHL incidence decreased after the COVID-19 pandemic.

ACE2, angiotensin-converting enzyme 2; COVID-19, coronavirus disease; MRI, magnetic resonance imaging; SARS-CoV-2, severe acute respiratory syndrome coronavirus 2; SSNHL, sudden sensorineural hearing loss; TMPRSS2, transmembrane protease serine2.
